# Ulcerative colitis therapeutic effects and pharmacokinetics of flexible quercetin nanocrystals prepared with geniposide assistance

**DOI:** 10.3389/fphar.2026.1873475

**Published:** 2026-09-22

**Authors:** Wei Lv, Haobo Liu, Haixian Feng, Huiqin Xue, Jiale Wen, Xinlei Fu, Xiaohong Zhang, Yongtai Xv, Huanyun Wang, Shibing Guo, Shikui Wu

**Affiliations:** 1 College of Medicine, Inner Mongolia Medical University, Hohhot, Inner Mongolia, China; 2 Pharmacy Department, Hohhot Mongolian Medicine of Traditional Chinese Medicine Hospital, Hohhot, China; 3 Nursing Department, Second Affiliated Hospital of Inner Mongolia Medical University, Hohhot, China

**Keywords:** bioavailability, geniposide, nanocrystals, quercetin, ulcerative colitis

## Abstract

**Objective:**

This study aimed to improve the oral bioavailability and therapeutic efficacy of quercetin (QUE) against ulcerative colitis (UC) by formulating flexible quercetin nanocrystals (QUE-NCs) with the assistance of geniposide (Gen).

**Methods:**

QUE-NCs were prepared via an antisolvent precipitation method leveraging the interaction between QUE and Gen. Therapeutic efficacy was evaluated in a dextran sulfate sodium (DSS)-induced UC mouse model using disease activity index (DAI), colon histopathology (H&E), and ELISA-based biochemical assays. Biosafety was assessed through serum biochemistry, organ indices, and histopathological examination. An HPLC method was developed and validated for quantifying QUE in rat plasma, followed by a pharmacokinetic study in Wistar rats.

**Results:**

QUE-NCs exhibited a flexible rod-like morphology and significantly alleviated UC symptoms, including weight loss, fecal occult blood, and colonic damage. Treatment with QUE-NCs reduced pro-inflammatory cytokines (TNF-α: 173.51 pg·mL^−1^; IL-6: 32.55 pg·mL^−1^), restored anti-inflammatory IL-10 levels (126.37 pg·mL^−1^), and suppressed oxidative stress markers (MPO: 579.95 pg·mL^−1^; MDA: 62.44 nmol·g^−1^; iNOS: 6.78 ng·mL^−1^). The nanocrystal formulation demonstrated excellent biosafety at the tested dose and significantly enhanced QUE’s oral bioavailability (294.42% relative to raw QUE).

**Conclusion:**

Gen-assisted formulation of QUE-NCs improves both the oral absorption and anti-UC efficacy of QUE, offering a promising nanostrategy for flavonoid-based therapeutic development.

## 1 Introduction

Ulcerative colitis (UC) is a chronic inflammatory disease characterized by diffuse inflammation of the colonic mucosa, with recurrent episodes often presenting as bloody diarrhea (Le Berre et al., 2023; Segal et al., 2021; Liu C. et al., 2022). The pathogenesis of UC involves genetic factors, lifestyle, and gut microbiota imbalance (Lin et al., 2020). Current treatments include biologics, aminosalicylates, corticosteroids, and immunomodulators (Segal et al., 2021; Cai et al., 2021; Zheng et al., 2023), but these therapies are associated with high infection rates and various complications (Zgheib et al., 2022; Mallick and Malik, 2022; Hayashi et al., 2024). Safe and effective therapeutics for UC have become a major research focus.

The flavonoid compound quercetin (QUE) has been extensively studied due to its diverse pharmacological activities, including antioxidant (Wang et al., 2023), antihypertensive (Zhang et al., 2023), antiviral (Shorobi et al., 2023), antidiabetic (Korkmaz and Dik, 2024), and anti-inflammatory (Kim et al., 2024) effects, demonstrating significant potential for applications in both food and pharmaceutical fields. In recent years, research on QUE for inflammatory diseases has advanced considerably. For instance, Gao (Gao et al., 2024) demonstrated that QUE restores intestinal barrier function in UC mice by inhibiting the cGAS-STING pathway in intestinal macrophages and rebalancing macrophage polarization. Wang (Wang et al., 2024) reported that QUE repairs intestinal barrier damage by activating aryl hydrocarbon receptor (AhR)-mediated tight junction protein expression. Khater (Khater et al., 2022) confirmed that QUE downregulates pro-inflammatory cytokines and oxidative stress biomarkers in colitis rats. QUE’s safety has been widely validated in animal models (Pinheiro et al., 2021); however, AST, ALT, BUN, and CREA levels were measured to assess the hepatorenal safety of QUE-NCs at administered doses (Gao et al., 2024; M et al., 2021).

The poor aqueous solubility of QUE limits its intestinal absorption, and extensive metabolism in the liver and intestinal epithelium converts QUE into less bioactive metabolites, further restricting its bioavailability (Kandemir et al., 2022; Aghababaei and Hadidi, 2023). In humans, QUE’s bioavailability is less than 1% (Fujimori et al., 2015). A study in Sprague-Dawley rats showed that 93% of orally administered QUE (50 mg·kg^-1^) was metabolized within 1 h, with only trace free QUE (0.9 μM) detected in plasma, primarily as glucuronide conjugates, followed by sulfate and sulfoglucuronide derivatives (Justino et al., 2004).

To address QUE’s bioavailability issues, structural modifications (Singh et al., 2018) or nanotechnological approaches—such as nanomicelles (Lu et al., 2022), composite nanoparticles (Zhou et al., 2021), nanoemulsions (Kumar and Ahad, 2023), and nanocrystals (NCs) (Manca et al., 2020)—have been explored. NCs are carrier-free colloidal delivery systems at the nanoscale (Junyaprasert and Morakul, 2015), offering advantages such as high stability, high drug loading, and enhanced saturation solubility (Li J. et al., 2021; Gülsün et al., 2013; Ran et al., 2022). In this study, QUE nanocrystals (QUE-NCs) were prepared via antisolvent precipitation and evaluated through *in vivo* pharmacokinetic and pharmacodynamic studies.

This study aimed to investigate the therapeutic efficacy of QUE-NCs against UC and their impact on QUE’s bioavailability. A dextran sulfate sodium (DSS)-induced UC model in BALB/c mice was used to compare the effects of QUE-NCs, QUE, and the positive control sulfasalazine (SASP). Additionally, the pharmacokinetic behaviors of QUE and QUE-NCs were assessed in Wistar rats following oral administration.

## 2 Materials and methods

### 2.1 Materials

QUE standard was purchased from Shanghai Yuanye Bio-Technology Co., Ltd. (O29HB199514). QUE was obtained from Bide Pharmatech (BUN332). Kaempferol standard was acquired from Shanghai Yuanye Bio-Technology Co., Ltd. (J17IB218678). HPLC-grade methanol was purchased from Thermo Fisher Scientific (241086). Fecal occult blood qualitative detection kit (R21607-300T, Shanghai Yuanye Bio-Technology Co., Ltd.). DSS was obtained from Dalian Meilun Biotechnology Co., Ltd. (J0730E). PBS buffer was purchased from Beijing Lanjieke Technology Co., Ltd. (BL601A). Sulfasalazine enteric-coated tablets were obtained from Shanghai Sine Tianping Pharmaceutical Co., Ltd. (H31020557). ELISA kits for tumor necrosis factor-α (TNF-α), interleukin-6 (IL-6), interleukin-10 (IL-10), myeloperoxidase (MPO), and inducible nitric oxide synthase (iNOS) were purchased from Shanghai Enzyme-linked Biotechnology Co., Ltd. Malondialdehyde (MDA) assay kit was acquired from Shanghai Enzyme-linked Biotechnology Co., Ltd. 4% paraformaldehyde fixative was purchased from Lanjieke Technology Co., Ltd. (24171851).

### 2.2 Animals

Twelve SPF-grade male Wistar rats (180–220 g), aged 8 weeks, were provided by the Animal Experiment Center of Inner Mongolia Medical University (license no. SYXK (Meng) 2020-0003). Thirty SPF-grade male BALB/c mice (25–27 g), aged 8 weeks, were purchased from SPF Biotechnology Co., Ltd. (license no. SCXK (Jing) 2024-0001). Prior to experiments, animals were acclimatized for 1 week under controlled conditions (21 °C ± 2 °C, 45%–65% humidity, 12 h light-dark cycle).

### 2.3 Experimental methods

#### 2.3.1 *In vitro* analysis of QUE-NCs

##### 2.3.1.1 Preparation of QUE-NCs

A 9.8 mg·mL^-1^ QUE ethanol solution and a geniposide aqueous solution were prepared. The solutions were mixed at a water/ethanol volume ratio of 12:1, resulting in a QUE/Gen mass ratio of 3.75:1 in the mixed solution. Ultrasonication was performed at 200 W for 5 min. The mixture was centrifuged at 16,000 rpm for 15 min to remove the supernatant, washed twice with distilled water, and finally redispersed in distilled water to obtain QUE-NCs.

##### 2.3.1.2 Characterization of QUE-NCs

A drop of QUE-NCs was placed on aluminum foil and air-dried. The sample was then adhered to a sample stage using conductive tape, sputter-coated with gold, and observed under SEM.

The QUE-NCs were diluted with distilled water to an appropriate concentration, and their particle size, PDI, and zeta potential were measured using a laser particle size analyzer. Measurements were performed in triplicate, and the average values were recorded.

##### 2.3.1.3 XRD analysis of QUE-NCs

X-ray diffraction (Cu-Kα radiation) with a graphite monochromator and scintillation counter was used to analyze QUE raw material and QUE-NCs.

##### 2.3.1.4 Storage stability of QUE-NCs

To evaluate the stability of QUE-NCs, the prepared samples were stored at room temperature and 4 °C in the dark. Particle size and polydispersity index (PDI) were measured daily for 7 days using a particle size analyzer. Measurements were performed in triplicate, and the average values were calculated. Observations were made for aggregation, turbidity, and precipitation.

##### 2.3.1.5 Saturation solubility of QUE-NCs

Excess QUE raw material and QUE-NCs were separately added to vials containing distilled water and shaken to prepare supersaturated solutions. The solutions were equilibrated in a 37 °C thermostatic shaker at 100 rpm for 24 h. After centrifugation at 16,000 rpm for 15 min, the supernatant was filtered through a 0.22 μm membrane. The filtrate was appropriately diluted, and the concentration was determined to calculate the saturation solubility.

#### 2.3.2 Pharmacodynamic experiments

##### 2.3.2.1 Animal grouping

Thirty male BALB/c mice were randomly divided into five groups (n = 6). The experimental protocol is detailed in Table 1. The total experimental period was 12 days. Except for the Control group, which received drinking water daily, all other groups were given 3% DSS in drinking water *ad libitum* for the first 7 days to establish the UC model. From day 6 of modeling, the respective drugs were administered via oral gavage. The Control group received an equivalent volume (0.1 mL·10 g^-1^) of normal drinking water. Gavage was performed at a fixed time daily for 7 consecutive days. To avoid microbial contamination, DSS solutions were replaced daily during modeling.

**TABLE 1:** Mouse grouping, drinking water, and dosing regimen.

Group	Drinking during modeling	Post-modeling water intake	Dosing regimen
Control	Normal drinking water	Normal drinking water	Pure water: 10 mL·kg^−1^·d^−1^
DSS	3% DSS drinking water	Normal drinking water	Pure water: 10 mL·kg^−1^·d^−1^
DSS + SASP	3% DSS drinking water	Normal drinking water	SASP: 527 mg·kg^−1^·d^−1^
DSS + QUE	3% DSS drinking water	Normal drinking water	QUE: 100 mg·kg^−1^·d^−1^
DSS + QUE-NCs	3% DSS drinking water	Normal drinking water	QUE-NCs: 100 mg·kg^−1^·d^−1^

The oral dose of 100 mg·kg^-1^ for both raw quercetin (QUE) and QUE-NCs was selected based on a comprehensive review of previous pharmacodynamic studies in rodent models of colitis, which consistently demonstrated significant therapeutic efficacy at this dose level without observable toxicity. To bridge to clinical relevance, we applied the U.S. Food and Drug Administration (FDA) guidance for converting animal doses to human equivalent doses (HED) based on body surface area. The HED for a 100 mg·kg^-1^ dose in mice is approximately 8.1 mg·kg^-1^ for a 60-kg human, which translates to a total daily dose of ∼486 mg. This falls well within the range of oral quercetin doses (500–1000 mg/day) commonly used in human nutritional supplementation and clinical trials, thereby supporting the translational potential of our findings. Sulfasalazine (SASP) was used as a positive control at a dose of 527 mg·kg^-1^. This dose was chosen based on its widespread use in the literature for DSS-induced colitis models and corresponds to the clinically recommended human-equivalent dose for treating ulcerative colitis, ensuring that our positive control reflects the practical clinical scenario.

##### 2.3.2.2 Tissue measurement and sampling

Body weight was recorded daily, and fecal occult blood was assessed. Twenty-four hours after the last dose, mice were fasted for 12 h, and blood was collected via orbital puncture for serum biochemical analysis. After cervical dislocation euthanasia, the abdominal cavity was opened to excise the heart, liver, spleen, lungs, and kidneys, which were weighed and preserved in 4% paraformaldehyde. The entire colon was removed, fat tissue was trimmed, and the colon was rinsed with cold 0.9% saline. The distal colon was transversely sectioned and stored in 4% paraformaldehyde for subsequent experiments.

##### 2.3.2.3 Detection indicators

###### 2.3.2.3.1 Disease activity index (DAI)

To assess inflammation severity, DAI was scored based on weight loss, stool consistency, and fecal occult blood (Table 2). Higher scores indicated more severe inflammation.

$$\text{DAI}=\left(\text{Weight loss score}+\text{Stool consistency score}+\text{Fecal blood score}\right)\;/\;3$$

**TABLE 2:** DAI scoring criteria (Jing et al., 2022).

Score	Weight loss	Stool consistency	Fecal blood
0	None	Normal	Negative (−)
1	1%–5%	Soft but formed	Positive (+)
2	6%–10%	Semi-loose	Slight visible blood (++)
3	11%–15%	Loose	Obvious visible blood (+++)
4	≥15%	Severe diarrhea	Gross bleeding (++++)

###### 2.3.2.3.2 Histological examination of mouse colon

Colon tissues were fixed, dehydrated, embedded, sectioned, stained (H&E), and imaged under a microscope.

##### 2.3.2.4 ELISA biochemical analysis of colon tissues

Colon tissues stored at −80 °C were thawed, homogenized in cold PBS (1:9 w/v), and centrifuged. Supernatants were analyzed for TNF-α, IL-6, IL-10, MPO, iNOS, and MDA using ELISA kits.

##### 2.3.2.5 Safety evaluation

###### 2.3.2.5.1 Serum biochemical analysis

Blood was collected from the mice in the Control and QUE-NCs groups via enucleation, with approximately 0.5 mL of blood drawn into heparin sodium-coated anticoagulant tubes. After standing at room temperature for 2 h, the samples were centrifuged at 3000 r·min-1 for 15 min (4 °C) to obtain the supernatant. Serum biochemical markers related to hepatic and renal function were analyzed using an automated biochemical analyzer. The measured parameters included alanine aminotransferase (ALT), aspartate aminotransferase (AST), blood urea nitrogen (BUN), and creatinine (CREA).

###### 2.3.2.5.2 Organ indices and histopathology

Heart, liver, spleen, lungs, and kidneys were weighed, and organ indices were calculated:

$$\text{Organ Index }\left(\%\right)=\left[\text{Organ Weight }\left(g\right)\;/\text{ Body Weight }\left(g\right)\right]\times 100\%$$

Tissues were fixed in 4% paraformaldehyde, sectioned, and stained for H&E analysis.

#### 2.3.3 Pharmacokinetic experiments

##### 2.3.3.1 HPLC conditions for QUE-NCs analysis

C_18_ column (250 mm × 4.6 mm, 5 μm); mobile phase: methanol-water (55:45, v/v); flow rate: 1.0 mL·min^-1^; detection wavelength: 375 nm; column temperature: 30 °C; injection volume: 10 μL. Method validation included specificity, linearity, LLOQ, precision, recovery, and stability Supplementary Figures S1, S2 and Supplementary Tables S1–S5.

##### 2.3.3.2 Plasma sample preparation

A 200 μL aliquot of rat plasma was placed in a 1.5 mL centrifuge tube, followed by the sequential addition of 50 μL of the internal standard kaempferol solution (25.00 μg·mL^−1^) and 350 μL of anhydrous ethanol (Shen et al., 2021). The mixture was vortexed for 90 s, centrifuged at 14,000 r·min^−1^ for 15 min, and the supernatant was transferred to a new 1.5 mL centrifuge tube. The supernatant was concentrated to dryness under nitrogen at 40 °C, and the residue was reconstituted in 50 μL of methanol. After vortexing for 90 s and centrifugation at 14,000 r·min^−1^ for 15 min, 10 μL of the supernatant was injected for analysis.

##### 2.3.3.3 Pharmacokinetic study design

Twelve male Wistar rats were randomly divided into two groups (n = 6). After fasting for 12 h (with free access to water), the rats were orally administered QUE-NCs or raw QUE at a dose of 150 mg**·**kg^−1^. Blood samples (0.5 mL) were collected from the jugular vein at 5 min, 10 min, 15 min, 0.5 h, 1 h, 2 h, 4 h, 6 h, 8 h, 12 h, and 24 h post-administration. The blood was placed in heparin sodium-coated centrifuge tubes, centrifuged at 3500 r·min^−1^ for 10 min (4 °C), and 200 μL of the supernatant was collected and stored at −80 °C for further analysis.

##### 2.3.3.4 Data analysis

The concentration of QUE in plasma was determined by HPLC, and the plasma concentration-time data were analyzed using DAS 2.0 software. The calculated pharmacokinetic parameters included: time to peak concentration (*t*_*max*_, h), maximum concentration (*C*_*max*_, μg·mL^−1^), elimination half-life (*t*_*1/2*_, h), area under the concentration-time curve from 0 to 24 h (*AUC*_*0-24*_, μg·mL^−1^ h), area under the concentration-time curve from 0 to infinity ( $\text{AU}C_{0-\infty }$ , μg·mL^−1^ h), and relative oral bioavailability (*F*_*r*_, %). The primary pharmacokinetic parameters were calculated using a non-compartmental model.

Data are presented as mean ± standard deviation (x̄ ± s). Statistical analysis was performed using SPSS 20.0 software, with intergroup comparisons conducted by t-test. A p-value <0.05 was considered statistically significant.

## 3 Results and discussion

### 3.1 Preparation of QUE-NCs

In classical crystal growth theory, new phases are formed by the sequential addition of monomers to isolated clusters (De Yoreo et al., 2015). In contrast, oriented attachment (OA), a non-classical crystal growth mechanism, involves the initial formation of loose primary particles that align through Brownian motion and eventually fuse into continuous crystals (Penn and Soltis, 2014). Based on experimental observations, the Gen-assisted growth of QUE crystals aligns with non-classical crystal growth theory.

During the Gen-assisted antisolvent precipitation process, the solubility of molecular QUE in ethanol decreases upon water addition, leading to the formation of QUE-NCs in the supersaturated solution. These QUE-NCs gradually align end-to-end, forming elongated QUE crystals with high aspect ratios. The (100) crystal plane of QUE interacts with water and Gen via hydrogen bonding, preventing close proximity between (100) planes and resulting in slender, flexible QUE crystals (Figure 1).

**FIGURE 1:**
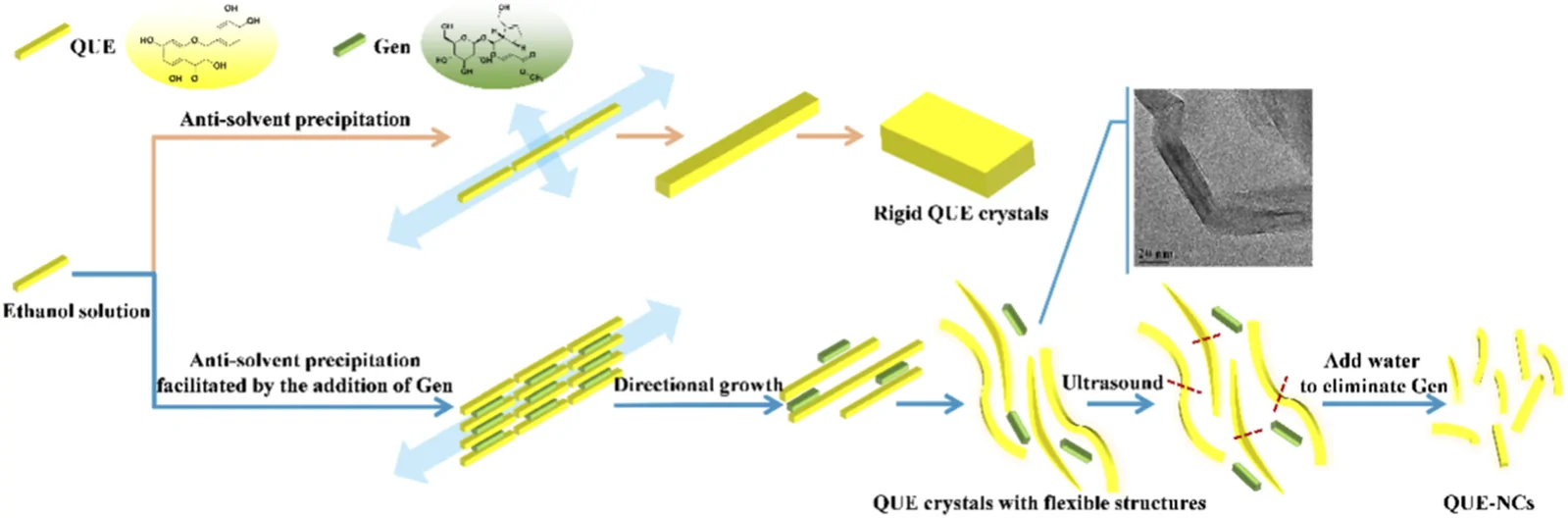
Schematic diagram of the preparation mechanism of QUE-NCs.

Natural crystallization is often difficult to control and exhibits poor reproducibility. However, ultrasonic cavitation significantly enhances the mixing efficiency among QUE, Gen, and water (Belca et al., 2019) while preventing aggregation of QUE-NCs (Prasad and Dalvi, 2020; Rengarajan and Gogate, 2025). Under ultrasonic conditions, the flexible QUE crystals further fracture into QUE-NCs.

### 3.2 Structural characterization of QUE-NCs

#### 3.2.1 Particle size, PDI, zeta potential, and XRD analysis

The QUE-NCs prepared by antisolvent precipitation (Figure 2a) exhibited a slender rod-like morphology with an average particle size of (178.2 ± 0.9) nm. The PDI was (0.200 ± 0.007), indicating good homogeneity. The zeta potential was (−34.4 ± 0.7) mV, with an absolute value exceeding 30 mV (Li JR. et al., 2021), demonstrating strong anti-aggregation properties and excellent stability.

**FIGURE 2:**
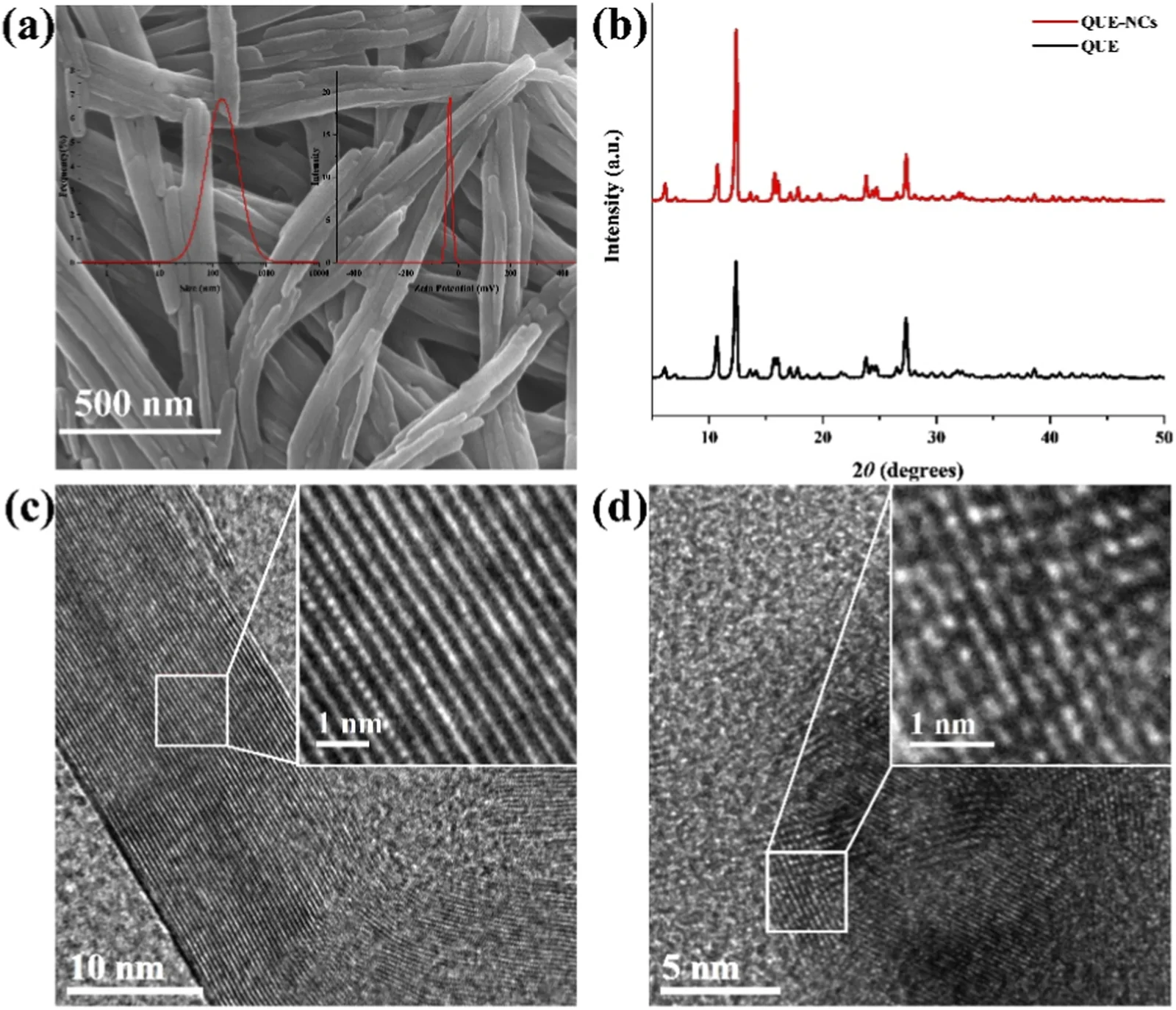
QUE-NCs **(a)** SEM image, particle size distribution, and zeta potential distribution **(b)** XRD pattern **(c,d)** HRTEM images.

The XRD pattern (Figure 2b) of raw QUE showed sharp diffraction peaks at 6.16°, 10.72°, 12.36°, 15.72°, 16.02°, 23.82°, and 27.30°, confirming its crystalline nature (Cheshmehnoor et al., 2022; Lu et al., 2023). The QUE-NCs prepared with Gen assistance exhibited diffraction peaks at nearly identical positions (6.18°, 10.72°, 12.40°, 15.80°, 16.10°, 23.82°, and 27.32°), with no additional peaks detected, confirming the absence of Gen residues. The sharp peaks indicated high crystallinity. Compared to raw QUE, the relative intensity of the 6.18° peak (corresponding to the (100) crystal plane) increased, while other peaks slightly decreased. The (100) plane is hydrophilic and grows via polar hydrogen bonding, suggesting Gen preferentially interacts with this facet. This preferential interaction, likely mediated by hydrogen bonding between the hydroxyl groups of Gen and the hydrophilic (100) plane of QUE, is the primary driving force for the observed crystal habit modification.

Despite Gen incorporation, QUE-NCs maintained high crystallinity, with well-ordered layered stacking stabilized by intermolecular hydrogen bonds and π-π interactions (Figure 2c). HRTEM (Figure 2d) revealed flexible structures attributed to Gen-induced lattice defects. The formation of these defects can be rationalized by the steric hindrance and localized lattice strain introduced by Gen molecules during the crystallization process, which disrupts the long-range order. These defects disrupted long-range order, explaining the reduced relative intensities of some XRD peaks.

#### 3.2.2 Storage stability evaluation of QUE-NCs

The short-term storage stability of QUE-NCs was assessed *in vitro*. Three batches of QUE-NCs suspensions were stored at room temperature and 4 °C, respectively. The particle size and PDI were measured daily for 7 consecutive days, with triplicate determinations performed for each measurement (Figures 3a,b).

**FIGURE 3:**
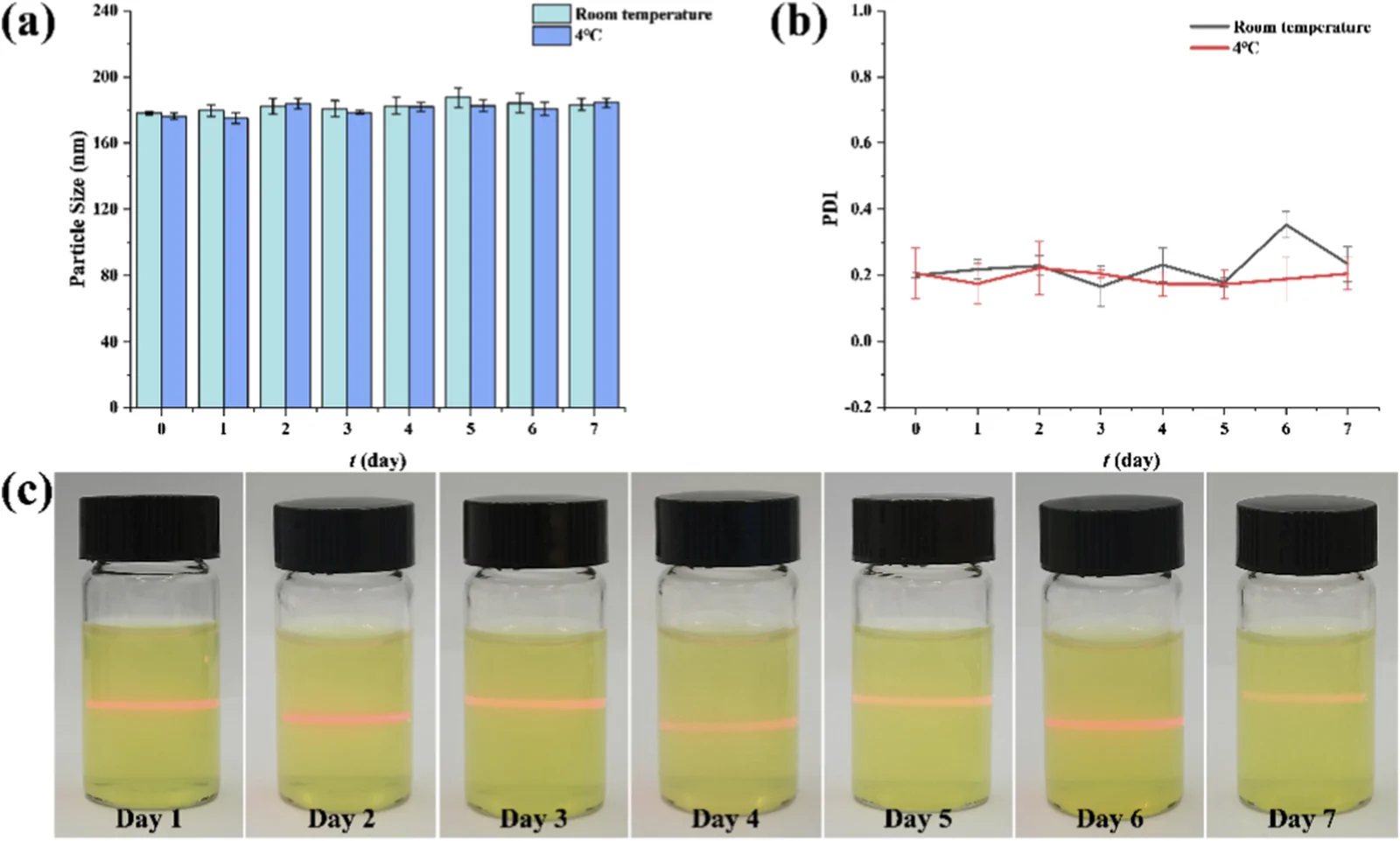
QUE-NCs under different conditions: **(a)** particle size, **(b)** PDI changes, **(c)** appearance of QUE-NCs during stability investigation.

Throughout the experimental period, no precipitation was observed in the suspensions (Figure 3c). Both particle size and PDI remained stable without significant variations, demonstrating good storage stability of the prepared QUE-NCs during the investigation period.

We acknowledge that the current stability assessment was limited to 7 days and did not include chemical stability monitoring (e.g., QUE degradation, Gen content) or crystallinity changes during storage. This short-term evaluation was specifically designed to ensure that the nanocrystals remained physically stable throughout the *in vivo* experimental period, during which all pharmacodynamic and pharmacokinetic studies were completed within days of formulation preparation. The physical stability data (particle size and PDI) confirmed that no significant aggregation or precipitation occurred within this timeframe, which is sufficient for our proof-of-concept study. Nevertheless, we fully agree that extended stability studies (e.g., 1–3 months) with comprehensive physicochemical characterization—including chemical stability, Gen content retention, and crystallinity assessment via XRD—are necessary for future translational development.

#### 3.2.3 Saturation solubility determination of QUE-NCs

The comparative results of saturation solubility between raw QUE and QUE-NCs are shown in Table 3. The saturation solubility of QUE-NCs was significantly higher (p < 0.001) than that of raw QUE, with QUE-NCs exhibiting 11.65-fold greater solubility. According to the Ostwald-Freundlich equation (Kesisoglou et al., 2007), drug saturation solubility increases with decreasing particle size. Solubility determines the extent to which drugs penetrate biological barriers and reach target sites (Ran et al., 2022; Imono et al., 2020), making it crucial for drug efficacy. QUE-NCs significantly enhanced QUE’s saturation solubility, potentially improving its *in vivo* performance.

**TABLE 3:** Saturation solubility results (x̄ ± s, n = 3).

Group	Saturation solubility (μg·mL^-1^)	RSD (%)
QUE	3.33 ± 0.04	1.23
QUE-NCs	38.81 ± 0.37***	0.96

*** denotes p < 0.001 vs. the raw QUE group.

### 3.3 Pharmacodynamic results

#### 3.3.1 Changes in body weight and DAI

DSS-induced clinical and histological responses resemble UC in humans. Its mechanism involves compromising colonic barrier integrity, allowing pro-inflammatory bacteria or luminal contents to infiltrate underlying tissues (Yang and Merlin, 2024; Lv et al., 2021). Thus, a UC model was established in BALB/c mice via *ad libitum* DSS consumption. UC typically presents with weight loss, diarrhea, and fecal occult blood—clinical markers reflecting disease severity.

As shown in Figure 4a, DSS induction caused significant weight loss in mice, while all treatments (SASP, QUE, QUE-NCs) promoted weight recovery, with QUE-NCs showing comparable efficacy to SASP by the final day (Figure 4b, p < 0.05 vs. DSS).

**FIGURE 4:**
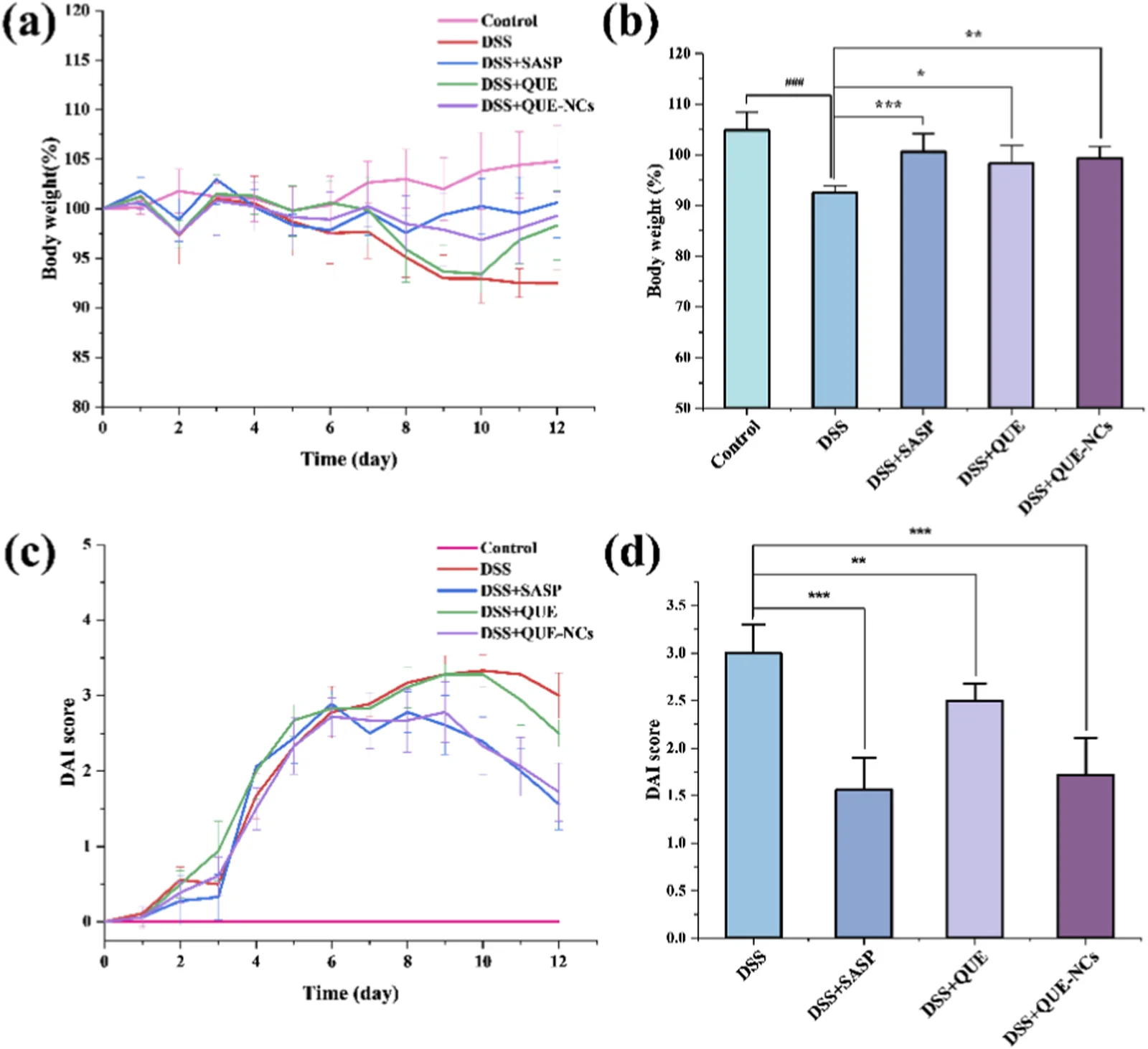
**(a)** Percentage changes in mouse body weight, **(b)** Percentage of mouse body weight on the last day of administration, **(c)** Changes in mouse DAI scores, **(d)** Mouse DAI scores on the last day of administration (n = 6). Note: Compared with Control group, ###p < 0.001; compared with DSS group, *p < 0.05, **p < 0.01, ***p < 0.001.

DAI scores progressively increased during DSS exposure and peaked by day 3 (Figure 4c). Following treatment, all groups exhibited reduced DAI scores compared to the DSS group (Figure 4d). QUE-NCs (1.72 ± 0.39) and SASP (1.56 ± 0.34) demonstrated the most pronounced reductions (p < 0.001 vs. DSS), while raw QUE showed a weaker effect (2.50 ± 0.18). Notably, QUE-NCs treatment completely resolved hematochezia and diarrhea, and improved the overall behavioral status of the mice.

#### 3.3.2 Macroscopic evaluation and histopathological analysis of colon

DSS challenge significantly shortened the colon length compared to the Control group (8.08 ± 0.56 vs. 10.18 ± 0.43 cm, p < 0.001). This shortening was effectively reversed by treatment with SASP (9.67 ± 0.37 cm), QUE (9.32 ± 0.69 cm), and QUE-NCs (9.52 ± 0.79 cm), with QUE-NCs and SASP restoring colon length to levels comparable to the Control group (Figures 5a,b, p > 0.05).

**FIGURE 5:**
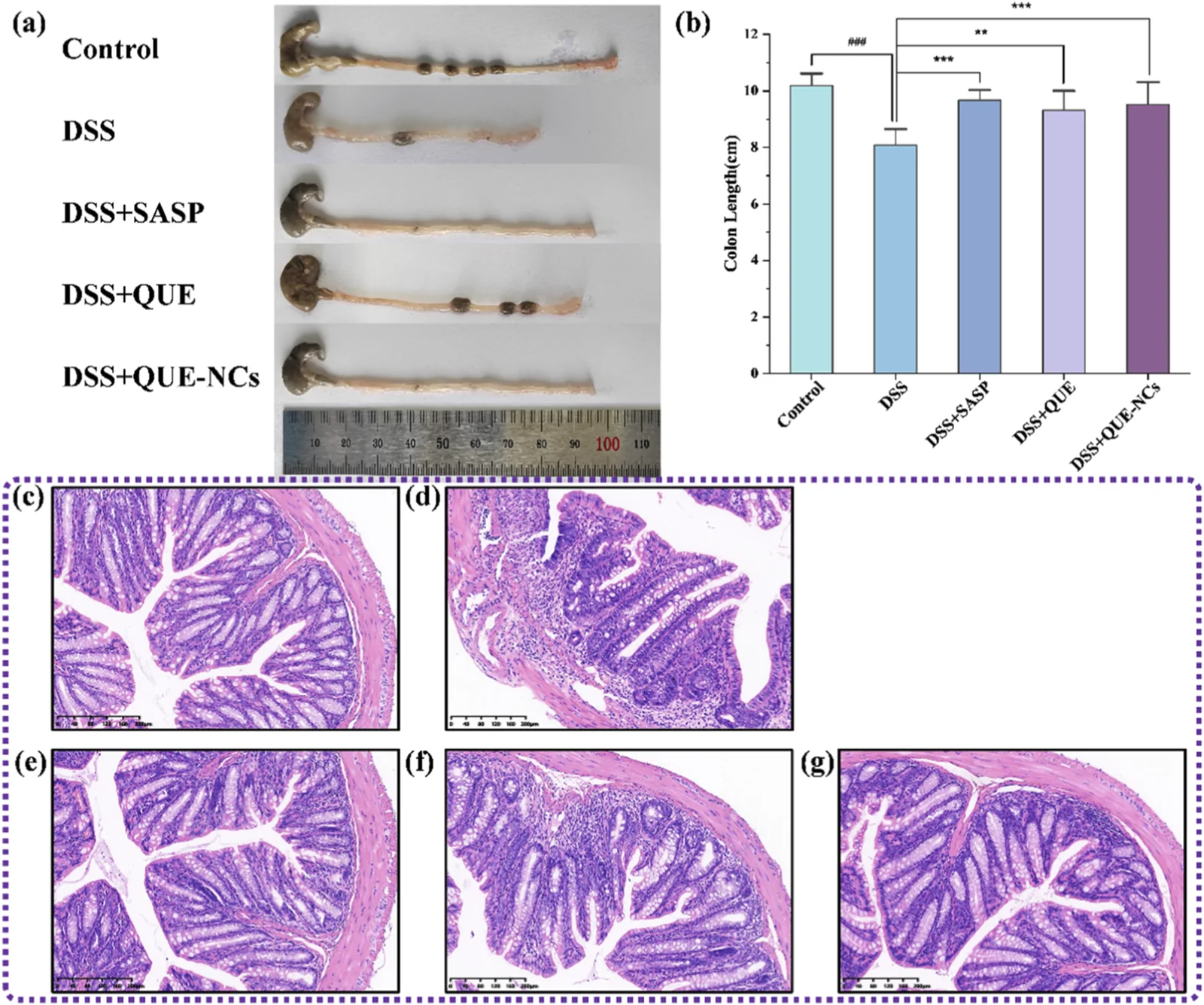
**(a)** Macroscopic appearance of mouse colons, **(b)** Quantitative analysis of colon length (n = 6), **(c)** Control group, **(d)** DSS group, **(e)** DSS + SASP group, **(f)** DSS + QUE group, **(g)** DSS + QUE-NCs group colon H&E staining (n = 6, scale bar 200 μm). Note: ###p < 0.001 vs. Control group; **p < 0.01, ***p < 0.001 vs. DSS group.

Histopathological examination (Figures 5c–g) revealed that DSS caused severe mucosal erosion, crypt distortion, goblet cell depletion, and extensive inflammatory infiltration across all colonic layers. QUE treatment partially restored crypt architecture with residual local inflammation. In contrast, both QUE-NCs and SASP groups exhibited well-preserved crypt structures, densely packed glands, and markedly reduced inflammatory infiltration, confirming effective mitigation of DSS-induced mucosal damage.

#### 3.3.3 TNF-α, IL-6, and IL-10 levels

The elevation of pro-inflammatory cytokines is a hallmark of DSS-induced ulcerative colitis. Therefore, the expression levels of TNF-α and IL-6 in mouse colon tissues were measured by ELISA. As shown in Figures 6a–c, DSS induction significantly elevated the pro-inflammatory cytokines TNF-α and IL-6 while suppressing the anti-inflammatory cytokine IL-10 in colon tissues (p < 0.001vs. Control). Oral administration of QUE-NCs, raw QUE, and SASP all significantly reversed these trends (p < 0.01 vs. DSS), with QUE-NCs showing superior efficacy in restoring IL-10 levels, comparable to that of SASP (p > 0.05). The IL-10 level in the QUE-NCs group was comparable to that in the SASP group (p > 0.05) and higher than that in the QUE group (p < 0.05), indicating that QUE-NCs significantly enhanced the therapeutic effect of raw QUE in restoring anti-inflammatory cytokine levels.

**FIGURE 6:**
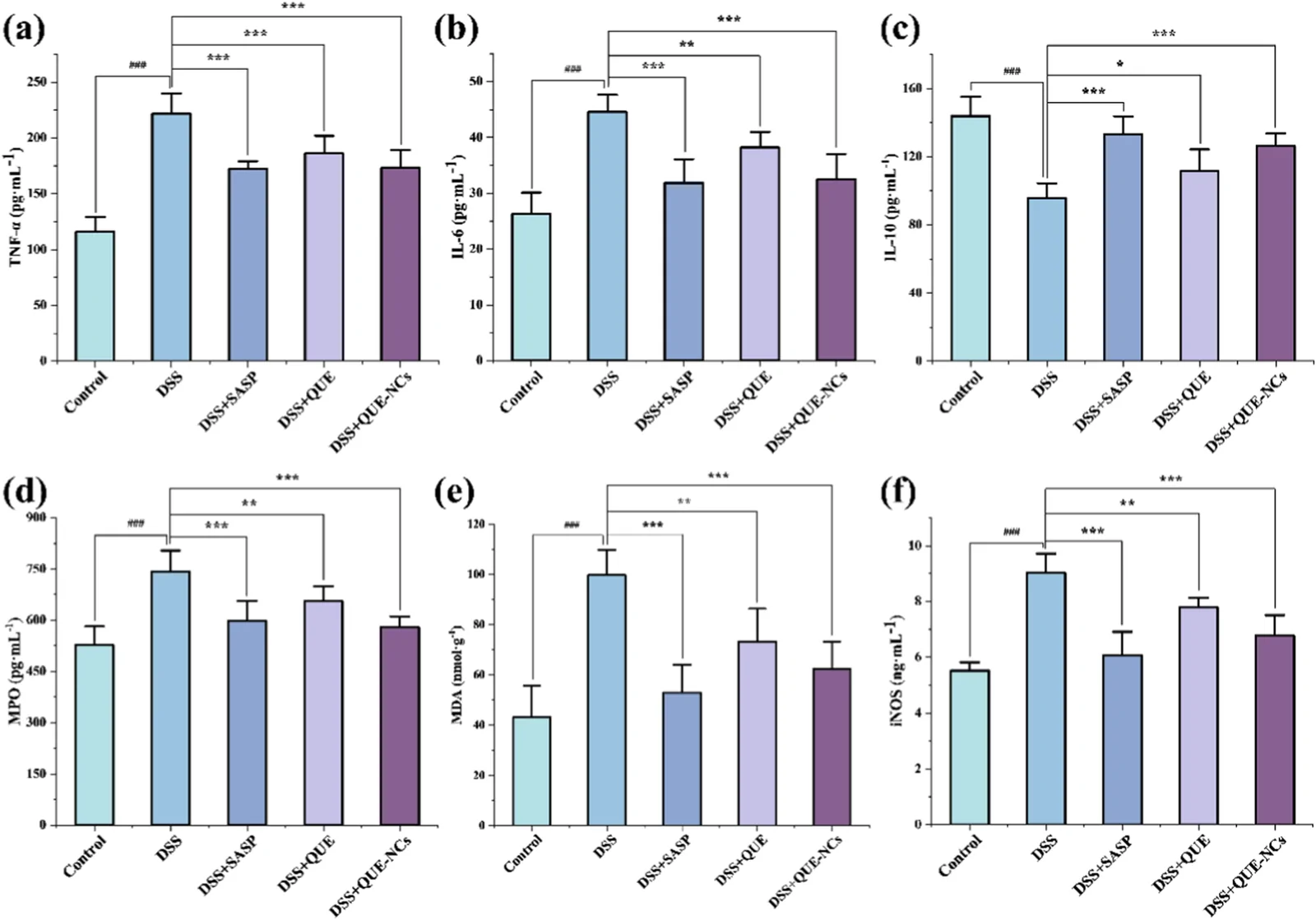
Expression levels in mouse colon of **(a)** TNF-α, **(b)** IL-6, **(c)** IL-10, **(d)** MPO, **(e)** MDA, and **(f)** iNOS (n = 6). Note: ### p<0.001 vs Control group; **p<0.01, ***p<0.001 vs DSS group.

These findings demonstrate that QUE-NCs effectively reduce the expression of pro-inflammatory cytokines (TNF-α, IL-6) and elevate the expression of the anti-inflammatory cytokine IL-10 in DSS-induced UC mice. Thus, the therapeutic efficacy of QUE-NCs against UC can be partially attributed to their superior anti-inflammatory activity. Although we did not perform dedicated immunophenotyping (e.g., T-cell subset analysis or macrophage polarization assays), the cytokine profiles we measured—including the suppression of pro-inflammatory TNF-α and IL-6 and the restoration of anti-inflammatory IL-10—indirectly reflect the immunomodulatory activity of QUE-NCs. These cytokines are key mediators of intestinal immune homeostasis, and their modulation is indicative of an anti-inflammatory immune shift. Future studies incorporating flow cytometric analysis of immune cell populations and immunohistochemical staining of colonic immune infiltrates would further elucidate the immunological mechanisms underlying the therapeutic effects of QUE-NCs.

#### 3.3.4 Oxidative stress in UC colon tissue

Oxidative stress has been identified as one pathogenic pathway in UC (Oliyaei et al., 2025), with UC patients exhibiting reduced antioxidant capacity, thus detection of oxidative stress biomarkers serves to validate therapeutic efficacy. MPO, MDA and iNOS are widely employed as oxidative damage biomarkers. Specifically, MPO is a heme protein secreted by phagocytes that serves as a marker for local leukocyte infiltration and inflammation (Moura et al., 2015). MDA, a key product of lipid peroxidation between oxygen free radicals and unsaturated fatty acids in cell membranes, causes significant damage to colonic mucosa and indirectly reflects oxygen free radical levels in UC patients’ colonic mucosa (Li et al., 2023). iNOS plays a crucial pathophysiological role in gastrointestinal conditions by generating peroxynitrite that induces tissue damage (Bhattacharyya et al., 2014).

Similarly, DSS challenge markedly increased the oxidative stress markers MPO, MDA, and iNOS, while all treatments significantly reduced their levels (Figures 6d–f, p < 0.01 vs. DSS). Notably, QUE-NCs again demonstrated comparable efficacy to SASP (p > 0.05) and outperformed raw QUE in suppressing MPO and iNOS (p < 0.05) confirming oxidative stress inhibition as a potential therapeutic mechanism of QUE-NCs in UC.

Oxidative stress further compromises intestinal barrier function, increasing permeability. Progressive inflammation enhances immune cell infiltration and reactive oxygen species (ROS) release in intestinal mucosa, with excessive ROS in the inflammatory microenvironment damaging biomolecules and intestinal epithelial cells (Blagov et al., 2023). Existing studies confirm QUE’s capacity to repair barrier damage through aryl hydrocarbon receptor activation (Wei et al., 2025).

The multifaceted therapeutic actions of QUE-NCs are mechanistically integrated in Figure 7. In summary, the enhanced oral bioavailability of QUE-NCs (as demonstrated in our pharmacokinetic data) allows sufficient concentrations of quercetin to reach the inflamed colonic mucosa, where it simultaneously: (i) suppresses the NF-κB-mediated pro-inflammatory cascade, thereby reducing TNF-α and IL-6 secretion while restoring IL-10 production; and (ii) scavenges reactive oxygen species and downregulates iNOS expression, thereby alleviating oxidative damage. This dual anti-inflammatory and anti-oxidative mechanism collectively restores mucosal integrity and alleviates UC symptoms. The pronounced efficacy of QUE-NCs correlates with their pharmacokinetic properties, warranting further investigation from a pharmacokinetic perspective.

**FIGURE 7:**
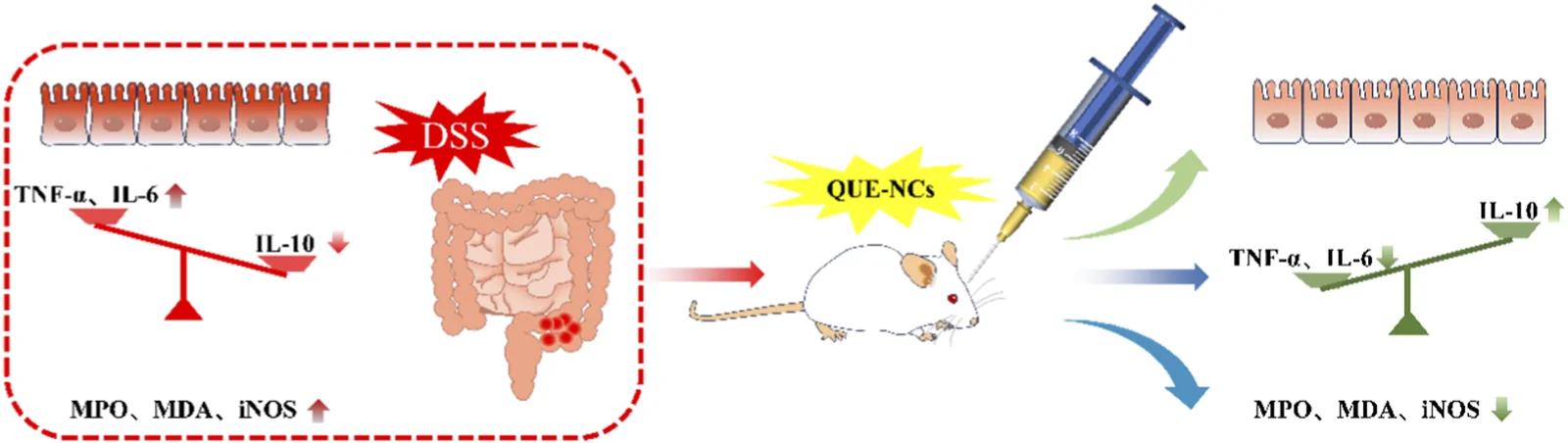
Schematic diagram summarizing the proposed therapeutic mechanisms of QUE-NCs in alleviating DSS-induced ulcerative colitis, integrating the anti-inflammatory (reduction of TNF-α, IL-6; elevation of IL-10) and anti-oxidative (reduction of MPO, MDA, iNOS) effects observed in Figure 6.

### 3.4 Safety evaluation results

#### 3.4.1 Serum biochemical parameters

Potential adverse effects were evaluated in QUE-NCs-treated mice using ALT and AST as hepatic function markers, and BUN and CREA as renal function indicators. Serum biochemical analysis (Figure 8) showed comparable levels between Control and QUE-NCs groups: ALT (42.939 ± 2.706 vs. 40.061 ± 1.095 U·L^-1^, p > 0.05), AST (157.274 ± 9.903 vs. 153.241 ± 8.725 U·L^-1^, p > 0.05), BUN (20.530 ± 0.616 vs. 19.503 ± 1.418 mg·dL^-1^, p > 0.05), and CREA (19.301 ± 0.535 vs. 20.310 ± 0.616 μmol·L^-1^, p > 0.05). These results demonstrate that oral administration of QUE-NCs at 100 mg·kg^-1^ caused no significant hepatorenal toxicity in mice.

**FIGURE 8:**
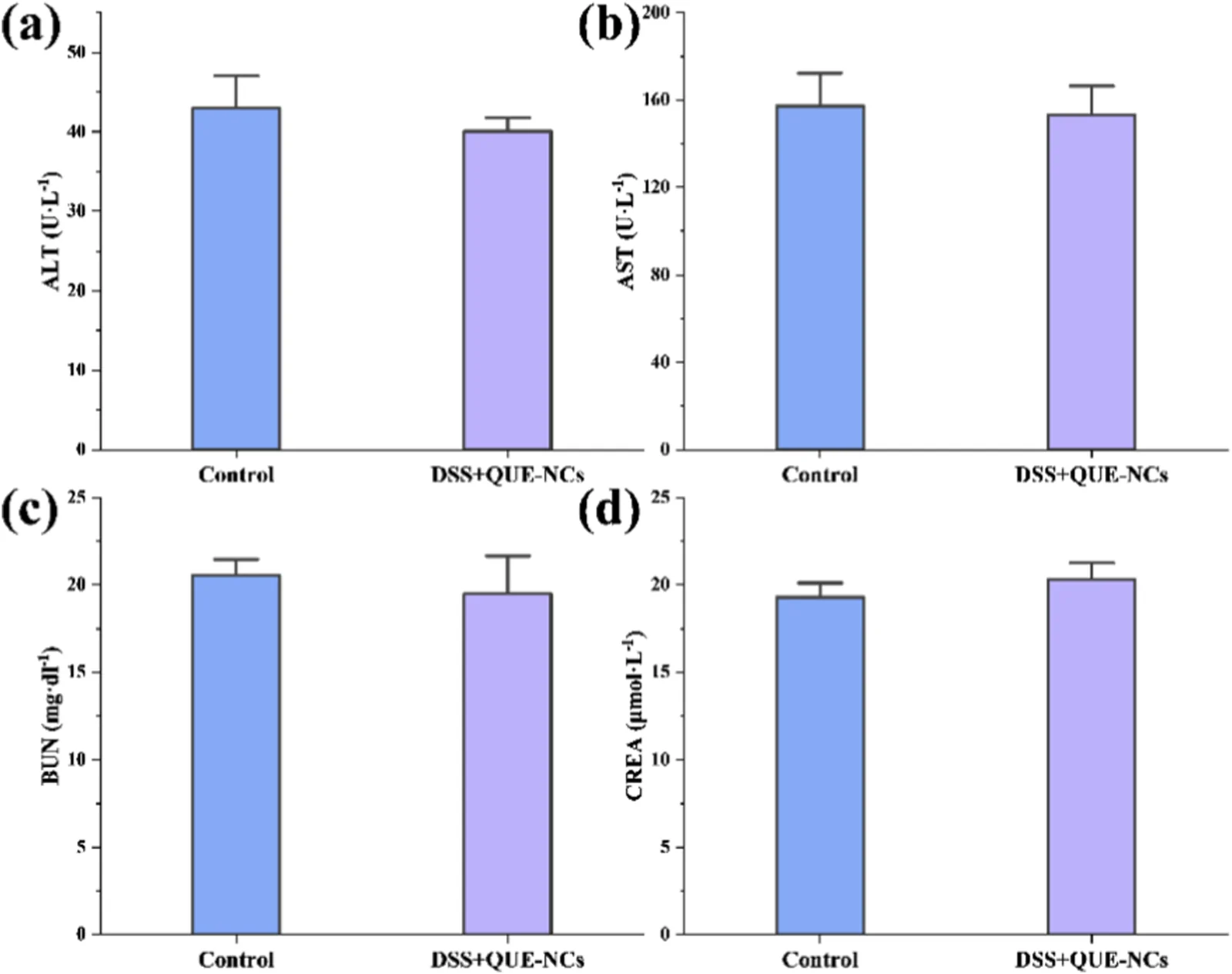
Serum levels of **(a)** ALT, **(b)** AST, **(c)** BUN, and **(d)** CREA in mice (n = 3).

#### 3.4.2 Organ indices and histopathological analysis

Statistical analysis of organ indices across groups (Table 4) revealed no significant differences between QUE-NCs and Control groups (p > 0.05).

**TABLE 4:** Mouse organ indices (x̄ ± s, n = 6).

Group	Heart	Liver	Spleen	Lungs	Kidneys
Control	0.60 ± 0.08	4.68 ± 0.27	0.30 ± 0.03	0.61 ± 0.05	1.60 ± 0.11
DSS + QUE-NCs	0.60 ± 0.08	4.36 ± 0.29	0.31 ± 0.03	0.70 ± 0.08	1.43 ± 0.12

As the spleen serves as a critical immune organ, DSS-induced immunopathology leads to compensatory splenomegaly (Wei et al., 2021), making spleen index comparison valuable for assessing inflammatory status. As shown in Supplementary Figure S3, DSS group exhibited significantly increased spleen index versus Control (p < 0.001), while QUE-NCs treatment markedly reduced this parameter (p < 0.001), outperforming raw QUE.

H&E staining of major organs (Figure 9) demonstrated preserved splenic architecture (distinct white/red pulp with intact capsule), normal pulmonary alveoli and bronchioles, and typical glomerular morphology without pathological alterations in other organs.

**FIGURE 9:**
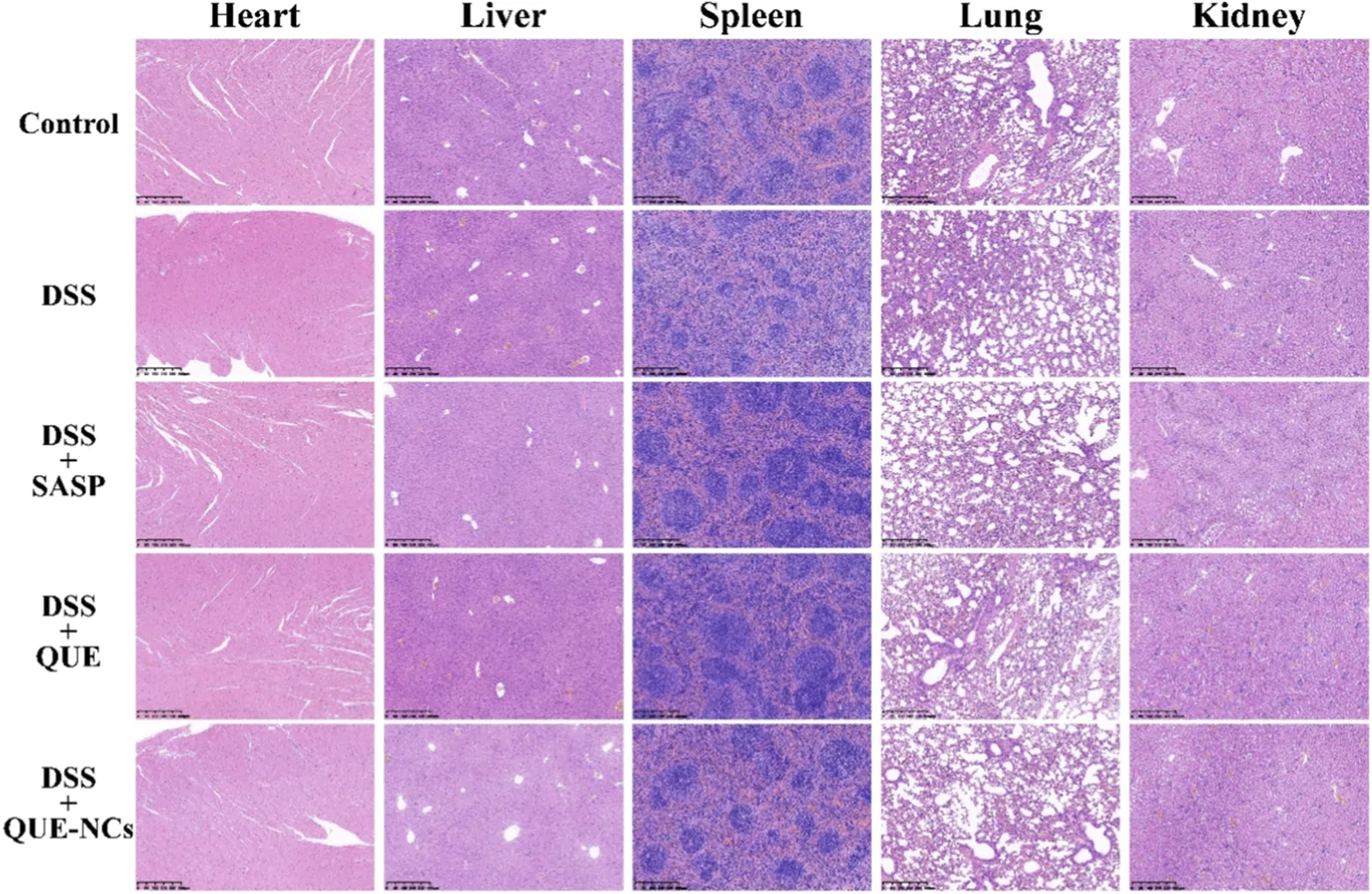
Histological sections of mouse organs (n = 3, scale bar 400 μm).

Combined organ indices, histopathological examination, and serum biochemical parameters collectively confirmed the safety profile of QUE-NCs at 100 mg·kg^-1^ dosage.

Regarding the safety comparison, the absence of a free QUE-treated group in the biosafety assessment warrants explanation. The safety profile of free quercetin has been extensively validated in numerous animal models and human studies at doses substantially higher than those used in our study (Pinheiro et al., 2021). Indeed, quercetin is classified as generally recognized as safe (GRAS) for food and supplement applications. Therefore, establishing the safety of QUE-NCs relative to the untreated (Control) group, rather than relative to free QUE, was considered sufficient to identify any nanocrystal formulation-specific toxicity. Our data confirmed that QUE-NCs at 100 mg·kg^-1^ did not cause hepatorenal toxicity or histopathological abnormalities, supporting their favorable safety profile.

### 3.5 Pharmacokinetic results

QUE exhibits poor absorption, extensive metabolism, and rapid elimination *in vivo*, resulting in relatively low oral bioavailability (Keranmu et al., 2022). Following oral administration, QUE is primarily absorbed by intestinal epithelial cells and undergoes phase II metabolism in the small intestine and liver (Zhu et al., 2024). The main metabolites detected in plasma are quercetin-3'-sulfate and quercetin-3-glucuronide (Dabeek and Marra, 2019), while free QUE exists in extremely low concentrations, necessitating acid hydrolysis for plasma sample pretreatment prior to QUE quantification in rat plasma.

Method validation demonstrated that the established HPLC method exhibited high precision and linearity, making it suitable for pharmacokinetic studies. Plasma drug concentrations at various time points were calculated using standard curves, and the resulting concentration-time profiles (Figure 10) revealed double-peak phenomena for both QUE and QUE-NCs, which is a typical pharmacokinetic signature often associated with enterohepatic recirculation for flavonoids, as previously reported in the literature (Zeng et al., 2016). We hypothesize that the observed secondary peaks for both formulations are also likely due to this recycling process; however, we acknowledge that direct evidence such as biliary excretion studies would be required for definitive confirmation.

**FIGURE 10:**
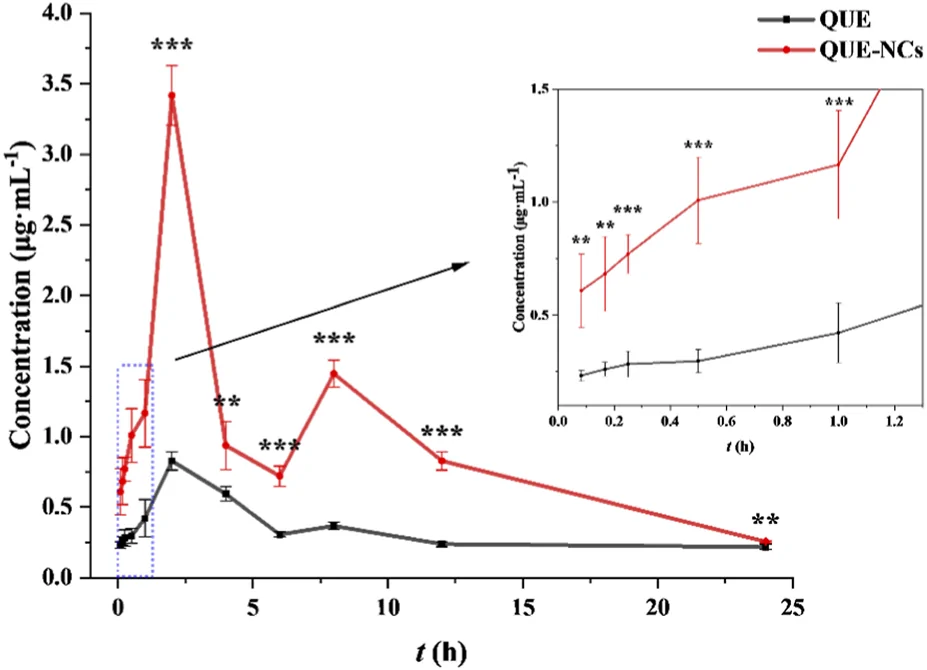
Plasma concentration-time curves of QUE-NCs and QUE (n = 6). Note: *p < 0.05, **p < 0.01, ***p < 0.001 vs. QUE group.

Pharmacokinetic parameters calculated using DAS 2.0 software (Table 5) showed that QUE-NCs achieved significantly higher C ∼ max∼ (4.12-fold, p < 0.001) and AUC∼0−∞ ∼ (2.94-fold, p < 0.001) compared to raw QUE, with a relative oral bioavailability of 294.42%.

**TABLE 5:** Pharmacokinetic parameters of QUE-NCs and QUE in rat plasma after oral administration (x̄ ± s, n = 6).

Parameter	QUE group	QUE-NCs group
*t*_max_ (h)	2.00 ± 0.00	2.00 ± 0.00
*C*_max_ (μg·mL^-1^)	0.83 ± 0.07	3.42 ± 0.21***
*t*_*1/2*_ (h)	6.10 ± 0.39	6.56 ± 0.19*
*AUC*_*0-24*_ (μg·mL^-1^·h)	7.88 ± 0.49	22.41 ± 1.48***
$AUC_{0-\infty }$ (μg·mL^-1^·h)	8.42 ± 0.51	24.79 ± 1.47***
*F*_*r*_ (%)	-	294.42

*p < 0.05, ***p < 0.001 vs. QUE group.

This enhanced bioavailability can be attributed to the reduced particle size, which increases dissolution surface area (Ma et al., 2022; Yu et al., 2022), improved mucus penetration and intestinal retention (Liu YF. et al., 2022), and enhanced cellular uptake of nanocrystals (Zhang et al., 2021; Tu et al., 2024). Furthermore, the rod-like morphology of QUE-NCs may confer superior mucus permeability and transepithelial transport compared to spherical particles (Guo et al., 2019; Zhu et al., 2022). Collectively, these physicochemical advantages of the nanocrystal formulation effectively overcome the bioavailability limitations of raw QUE.

We note that our discussion regarding the potential improvement of intestinal barrier function by QUE-NCs is based on mechanistic inference from the literature rather than direct experimental measurements in this study. Previous studies have demonstrated that quercetin activates aryl hydrocarbon receptor (AhR) to upregulate tight junction proteins such as ZO-1 and occludin, thereby repairing intestinal barrier damage in UC models (Wang et al., 2024; Wei et al., 2025). Given that QUE-NCs delivered significantly higher quercetin exposure (294.42% bioavailability) to the colonic tissues, it is reasonable to infer that similar barrier-protective effects may contribute to the observed therapeutic outcomes. However, we acknowledge that direct immunohistochemical or Western blot analysis of tight junction proteins (e.g., ZO-1, occludin, claudin-1) would provide definitive evidence. This represents a valuable direction for future mechanistic studies.

We acknowledge that direct measurement of quercetin and its metabolites in colonic tissue would provide more definitive evidence for the relationship between nanocrystal formulation, intestinal exposure, and therapeutic efficacy. While we did not perform such tissue distribution analyses in the present study, our pharmacokinetic data (significantly increased systemic exposure) and pharmacodynamic data (markedly improved colonic anti-inflammatory and anti-oxidative effects) collectively support a strong correlation between enhanced bioavailability and improved efficacy. The improved solubility and rod-like morphology of QUE-NCs further favor intestinal mucus penetration and local drug accumulation, as previously reported (Guo et al., 2019; Zhu et al., 2022). Nevertheless, direct tissue concentration measurement represents an important direction for future mechanistic studies to fully establish the site-specific exposure–response relationship.

## 4 Conclusion

In this study, QUE-NCs prepared by antisolvent precipitation were evaluated through pharmacokinetic and pharmacodynamic analyses. Pharmacokinetic results demonstrated that QUE-NCs exhibited 4.12-fold higher Cmax, 2.94-fold greater ${\text{AUC}}_{0-\infty }$ , and 294.42% relative bioavailability compared to raw QUE, confirming enhanced oral bioavailability. Pharmacodynamic studies revealed that QUE-NCs significantly alleviated DSS-induced UC symptoms, including weight loss, colon shortening, mucosal damage, elevated pro-inflammatory cytokines, reduced anti-inflammatory factors, and colonic oxidative stress. Overall, QUE-NCs demonstrate promising therapeutic potential for UC with favorable biosafety. We acknowledge a limitation of this study: pharmacodynamic evaluations were performed in BALB/c mice, while pharmacokinetic studies were conducted in Wistar rats. This cross-species design was adopted for pragmatic reasons—the DSS-induced colitis model is most established and reproducible in BALB/c mice, whereas pharmacokinetic studies require serial blood sampling, which is more feasible in the larger Wistar rat. Although differences in metabolism, gut microbiota, and immune responses between these species may affect the absolute values of PK and PD parameters, the core conclusion—that nanocrystal formulation enhances oral bioavailability and thereby improves anti-colitis efficacy—remains robust and is supported by the consistent trends observed across both models.

## Data Availability

The original contributions presented in the study are included in the article/Supplementary Material, further inquiries can be directed to the corresponding authors.
