# Supplementary material for: Ulcerative colitis therapeutic effects and pharmacokinetics of flexible quercetin nanocrystals prepared with geniposide assistance
Source: Front Pharmacol. 2026 Sep 22;17:1873475. doi: 10.3389/fphar.2026.1873475 (PMC13638574; doi:10.3389/fphar.2026.1873475)
Supplement: Supplementary file 1 [file Supplementaryfile1.docx]

**Supporting Information**

**1 Preparation of reference standard and internal standard solutions**

Preparation of reference standard solution: An appropriate amount of QUE reference standard was accurately weighed and transferred to a 25 mL brown volumetric flask. Methanol was added to dissolve and dilute to the mark, yielding a QUE reference standard solution with a concentration of 100 μg·mL⁻¹. The solution was stored at 4 °C for subsequent use.

Preparation of internal standard solution: An appropriate amount of kaempferol reference standard was accurately weighed and transferred to a 25 mL brown volumetric flask. Methanol was added to dissolve and dilute to the mark, yielding an internal standard stock solution with a concentration of 100 μg·mL⁻¹. A portion of this solution was further diluted with methanol to obtain an internal standard working solution with a concentration of 25 μg·mL⁻¹. The solution was stored at 4 °C for subsequent use.

**2 Validation of HPLC method**

**2.1 Specificity evaluation**

Blank rat plasma samples spiked with 50 μL internal standard solution and 50 μL QUE reference standard solution, and plasma samples collected 0.25 h after oral administration of QUE-NCs (150 mg·kg⁻¹) with 50 μL internal standard solution were processed and analyzed by HPLC (Figure S1). The retention times were approximately 8.29 min for QUE and 12.16 min for the internal standard. QUE and the internal standard showed complete separation without interference from endogenous substances, demonstrating good method specificity.

**Fig. S1** (a) Blank plasma + QUE reference standard solution + internal standard solution;

(b) Drug-containing plasma + internal standard solution;

(c) Blank plasma (1: QUE; 2: Internal standard)

**2.2 Linearity and lower limit of quantification (LLOQ)**

A series of plasma calibration standards containing QUE at concentrations of 0.25, 0.50, 1.00, 2.50, 5.00, 10.00, and 12.50 μg·mL⁻¹ were prepared by adding appropriate volumes of QUE reference standard solution to 200 μL blank plasma. After sample pretreatment and chromatographic analysis, peak areas were recorded. The calibration curve was constructed by plotting the peak area ratio of QUE to internal standard (Y) versus QUE concentration (X), yielding the regression equation Y = 0.1368X - 0.0255 (R² = 0.9997, Figure S2). The results demonstrated good linearity over the concentration range of 0.25-12.50 μg·mL⁻¹, with an LLOQ of 0.17 μg·mL⁻¹.

**Fig. S2** Standard curve of QUE in rat plasma

**2.3 Precision evaluation**

Blank rat plasma (200 μL) was spiked with QUE reference standard and internal standard solutions to prepare plasma samples containing QUE at concentrations of 0.50, 2.50, and 10.00 μg·mL⁻¹. After sample pretreatment and chromatographic analysis (**Table S1**), each concentration level was analyzed in triplicate within one day to determine intra-day precision (RSD = 7.06%, 5.39%, and 9.65%, respectively). The inter-day precision was assessed by analyzing samples over three consecutive days (RSD = 7.28%, 9.11%, and 6.63%, respectively).

**Table S1** Precision of QUE in rat plasma ($\bar{x}$± *s*，*n* = 3)

| Preparation concentration (μg·mL^-1^) | Intra-day precision | | Daytime precision | |
| --- | --- | --- | --- | --- |
|  | Determination of concentration(μg·mL^-1^) | *RSD* (%) | Determination of concentration(μg·mL^-1^) | *RSD* (%) |
| 0.5 | 0.55 ± 0.04 | 7.06 | 0.55 ± 0.04 | 7.28 |
| 2.5 | 2.30 ± 0.12 | 5.39 | 2.25 ± 0.20 | 9.11 |
| 10.0 | 10.10 ± 0.97 | 9.65 | 10.22 ± 0.67 | 6.63 |

**2.4** **Investigation of Recovery Rate**

Blank rat plasma was spiked with internal standard and QUE reference standard solutions at low, medium, and high concentrations (0.50, 2.50, and 10.00 μg·mL⁻¹, respectively). Following sample pretreatment and chromatographic analysis, recovery rates were calculated as the ratio of measured to theoretical concentrations (**Table S2**). The recoveries were 103.65 ± 2.76%, 98.11 ± 3.91%, and 101.24 ± 3.14% for the three concentration levels, respectively.

**Table S2** Recovery of QUE in rat plasma($\bar{x}$± *s*，*n* = 5)

| Preparation concentration (μg·mL^-1^) | Determination of concentration(μg·mL^-1^) | Average recovery rate (%) | *RSD* (%) |
| --- | --- | --- | --- |
| 0.5 | 0.52 ± 0.01 | 103.65 ± 2.76 | 2.66 |
| 2.5 | 2.45 ± 0.10 | 98.11 ± 3.91 | 3.99 |
| 10.0 | 10.12 ± 0.31 | 101.24 ± 3.14 | 3.11 |

**2.5** **Stability evaluation**

Blank rat plasma (200 μL) was spiked with QUE reference standard and internal standard solutions to prepare quality control samples at concentrations of 0.50, 2.50, and 10.00 μg·mL⁻¹. The samples were divided into three groups: (1) stored at room temperature for 12 h, (2) stored at -20°C for 48 h, and (3) subjected to three freeze-thaw cycles (-20°C to room temperature). Following sample pretreatment and chromatographic analysis, the stability results (Tables S3-S5) demonstrated RSD values <5% for all conditions, indicating good stability under the tested storage conditions.

**Table S3** Stability of samples stored at room temperature for 12 h ($\bar{x}$± *s*，*n* = 5)

| Theoretical concentration /μg·mL^-1^ | Determination of concentration /μg·mL^-1^ | *RSD*/% |
| --- | --- | --- |
| 0.5 | 0.58 ± 0.03 | 4.69 |
| 2.5 | 2.52 ± 0.08 | 3.13 |
| 10.0 | 9.87 ± 0.26 | 2.60 |

**Table S4** Stability of samples stored at -20°C for 48 h ($\bar{x}$± *s*，*n* = 5)

| Theoretical concentration /μg·mL^-1^ | Determination of concentration /μg·mL^-1^ | *RSD*/% |
| --- | --- | --- |
| 0.5 | 0.51 ± 0.02 | 4.06 |
| 2.5 | 2.48 ± 0.12 | 4.66 |
| 10.0 | 10.09 ± 0.19 | 1.92 |

**Table S5** Stability of samples after three freeze-thaw cycles ($\bar{x}$± *s*，*n* = 5)

| Theoretical concentration /μg·mL^-1^ | Determination of concentration /μg·mL^-1^ | *RSD*/% |
| --- | --- | --- |
| 0.5 | 0.57 ± 0.02 | 3.95 |
| 2.5 | 2.51 ± 0.08 | 3.20 |
| 10.0 | 10.06 ± 0.23 | 2.26 |

**3. Spleen index of mice**

**Fig. S3** Spleen index of mice (n = 6).

Note: ### p<0.001 vs Control group; **p<0.01, ***p<0.001 vs DSS group.

**4. Main Experimental Reagents and Materials**

| Reagent Name | Batch Number | Manufacturer |
| --- | --- | --- |
| Fecal Occult Blood Qualitative Test Reagent | R21607-300T | Shanghai Yuanye Biotechnology Co., Ltd. |
| Quercetin | BUN332 | Bide Pharmatech Co., Ltd. |
| Dextran Sulfate Sodium Salt | J0730E | Dalian Meilun Biotechnology Co., Ltd. |
| PBS Phosphate Buffered Saline | BL601A | Beijing Lanjieke Technology Co., Ltd. |
| Sulfasalazine Enteric-Coated Tablets | H31020557 | Shanghai Xinyi Tianping Pharmaceutical Co., Ltd. |
| Mouse Tumor Necrosis Factor α ELISA Kit | ml002095E | Shanghai Enzyme-linked Biotechnology Co., Ltd. |
| Mouse Interleukin 10 ELISA Kit | ml037873E | Shanghai Enzyme-linked Biotechnology Co., Ltd. |
| Mouse Interleukin 6 ELISA Kit | ml098430E | Shanghai Enzyme-linked Biotechnology Co., Ltd. |
| Mouse Myeloperoxidase ELISA Kit | ml002070E | Shanghai Enzyme-linked Biotechnology Co., Ltd. |
| Mouse Inducible Nitric Oxide Synthase ELISA Kit | ml057773E | Shanghai Enzyme-linked Biotechnology Co., Ltd. |
| Malondialdehyde Biochemical Assay Kit | ml092884 | Shanghai Enzyme-linked Biotechnology Co., Ltd. |
| 4% Paraformaldehyde Fixative Solution | 24171851 | Lanjieke Technology Co., Ltd. |
